# Safety of fondaparinux to prevent venous thromboembolism in Japanese patients undergoing colorectal cancer surgery: a multicenter study

**DOI:** 10.1007/s00595-014-0911-7

**Published:** 2014-05-20

**Authors:** Taishi Hata, Masayoshi Yasui, Kohei Murata, Masaki Okuyama, Masayuki Ohue, Masataka Ikeda, Shigeyuki Ueshima, Kotaro Kitani, Junichi Hasegawa, Hiroshi Tamagawa, Makoto Fujii, Atsushi Ohkawa, Takeshi Kato, Shunji Morita, Takayuki Fukuzaki, Tsunekazu Mizushima, Mitsugu Sekimoto, Riichiro Nezu, Yuichiro Doki, Masaki Mori, Multi-Center Clinical Study Group of Osaka, Colorectal Cancer Treatment Group (MCSGO)

**Affiliations:** 1 Department of Gastroenterological Surgery, Graduate School of Medicine, Osaka University, Osaka, Japan; 2 Department of Surgery, Kaizuka City Hospital, Kaizuka, Japan; 3 Department of Surgery, Suita Municipal Hospital, Suita, Japan; 4 Department of Surgery, Higashiosaka City General Hospital, Higashiōsaka, Japan; 5 Department of Surgery, Osaka Medical Center for Cancer and Cardiovascular Diseases, Osaka, Japan; 6 Department of Surgery, National Hospital Organaization, Osaka National Hospital, 2-1-14 Hoenzaka, Chuo-ku, Osaka, 540-0006 Japan; 7 Department of Surgery, Osaka Police Hospital, Osaka, Japan; 8 Department of Surgery, Nara Hospital, Kinki University Faculty of Medicine, Osaka, Japan; 9 Department of Surgery, Osaka Rosai Hospital, Osaka, Japan; 10 Department of Surgery, Osaka General Medical Center, Osaka, Japan; 11 Department of Surgery, Osaka Kouseinenkin Hospital, Osaka, Japan; 12 Department of Surgery, Higashi Takarazuka Satoh Hospital, Takarazuka, Japan; 13 Department of Surgery, Kansai Rosai Hospital, Amagasaki, Japan; 14 Department of Surgery, Toyonaka Municipal Hospital, Toyonaka, Japan; 15 Department of Surgery, Ikeda Municipal Hospital, Ikeda, Japan; 16 Department of Surgery, Nishinomiya Municipal Central Hospital, Nishinomiya, Japan

**Keywords:** Venous thromboembolism, Prophylaxis, Colorectal cancer patients, Fondaparinux, Japan

## Abstract

**Purpose:**

To investigate the safety and efficacy of fondaparinux (FPX) for venous thromboembolism (VTE) prophylaxis in Japanese patients undergoing colorectal cancer surgery.

**Methods:**

The subjects of this multicenter, open-label, prospective observational study were patients undergoing resection of the colon/rectum for colorectal cancer. All patients were given FPX 2.5 or 1.5 mg by subcutaneous injection, once daily for 4–8 days, starting 24 h after surgery. The primary endpoint was any major bleeding event and the secondary endpoint was any symptomatic VTE event.

**Results:**

Between February 2009 and December 2010, 619 patients from 23 institutions were enrolled in this study. The median duration of FPX prophylaxis was 4 days. The incidence of major bleeding was 0.81 % [5/619, 95 % confidence interval (CI) 0.3–1.9] and the incidence of minor bleeding was 9.5 % (59/619, 95 % CI 7.3–12.1). There was no fatal bleeding or symptomatic VTE. Multivariable analysis revealed the following to be risk factors for bleeding events: preoperative platelet count <15 × 10^4^/µl [odds ratio (OR) 4.521], male sex (OR 2.078), and blood loss during surgery <50 ml (OR 2.019).

**Conclusion:**

The administration of 2.5/1.5 mg FPX 24 h after colorectal cancer surgery is safe and effective.

## Introduction

Venous thromboembolism (VTE) is a common complication of abdominal surgery [1]. According to a recent study in Japan, VTE occurred in 24.3 % of abdominal surgery patients, including one with symptomatic pulmonary embolism (PE) [2]. A comparable incidence has been reported in Western countries [3]. These findings support active VTE prophylaxis after abdominal surgery.

Two randomized studies have been conducted in Japan, using two different pharmacological agents to prevent VTE: enoxaparin and fondaparinux (FPX). In both studies, pharmacological VTE prophylaxis proved more effective than mechanical prophylaxis, such as intermittent pneumatic compression, alone and did not increase bleeding events [4, 5]. However, these two Japanese prospective studies assessed only the safety of these pharmacological agents in 187 patients. Moreover, both studies comprised patients undergoing gastroenterological, gynecological, or urological procedures, so that patient heterogeneity did not allow stratification of bleeding risk for any specific condition, such as major cancer surgery requiring lymph node dissection and bowel anastomosis.

Postoperative bleeding is a concern for patients receiving pharmacological prophylaxis for VTE. Bleeding is easily detectable but can cause serious complications. On the other hand, VTE is clinically silent unless actively searched for and seldom causes serious conditions; however, once discovered, VTE requires many medical resources for treatment, which is why it is important to establish safe VTE prophylaxis. Bleeding complications after surgery sometimes depend on the type of surgical procedure, such as whether it is open or laparoscopic and if there is bowel anastomosis. Precise analysis of bleeding events can enable surgeons to use pharmacological agents and to better prepare for bleeding events.

This prospective study evaluates the safety of FPX for the prevention of VTE in Japanese patients undergoing colorectal cancer surgery, using the dosage regimen already approved for abdominal surgery in this country.

## Methods

We conducted a multicenter, open-label, observational study at 23 affiliated medical institutions between February 2009 and December 2010. This study was approved by the appropriate institutional review boards and undertaken according to the ethical principles stated in the Declaration of Helsinki (1964).

### Study protocol and patient recruitment

The inclusion criteria for this study were that patients underwent elective surgery for colorectal malignancy and that they gave written informed consent. Exclusion criteria were as follows: active bleeding; thrombocytopenia, defined as a platelet count of <10 × 10^4^/µL; disorders associated with an increased risk of bleeding, such as gastrointestinal tract ulcers, diverticulitis, colitis, acute bacterial endocarditis, severe uncontrolled hypertension, or severe uncontrolled diabetes mellitus; severe hepatic dysfunction (Child C); a known history of hypersensitivity to unfractionated heparins, low-molecular-weight heparins, or heparinoids; a history of intracranial bleeding; a history of surgical intervention of the central nervous system or ocular surgery within the past 3 months; unexpected bleeding or difficulty of hemostasis during surgery; severe renal dysfunction, defined as a creatinine clearance of <20 ml/min; a history of major orthopedic, abdominal, or cardiovascular surgery within the past 3 months; any treatment with anticoagulants, dextran, thrombolytics, or antiplatelet agents within the past week; clinical signs of VTE; a preoperative D-dimer >1 µg/ml or twice the institutional limit; a history of arterial thromboembolism; drug abuse or alcohol dependence; another elective surgical intervention during the study period; pregnancy or lactation; several attempts at, or bleeding during, epidural catheter insertion; and being deemed by the attending physician as unfit for the study. Because patients with high D-dimer levels might be at risk of thrombosis preoperatively, they were excluded from the study, and VTE prophylaxis was left to the discretion of the physician.

The study protocol included approved use of epidural anesthesia when necessary. The catheter had to be removed after 20 h of FPX administration, and FPX was required to be administered for 2 h after catheter withdrawal.

### VTE prophylaxis

FPX administration was started 24 h after surgery, once hemostasis was established, following the Japanese regimen for VTE prevention. FPX (2.5 or 1.5 mg) was given once daily for 4–8 days. Mechanical VTE prophylaxis, such as intermittent pneumatic compression (IPC), elastic stockings (ES), and elastic bandage (EB), was not prohibited by the protocol, with their use and duration left to the discretion of the investigators or institutions. An FPX dose of 1.5 mg was administered when creatinine clearance was <50 ml/min, body weight was <40 kg, or age was ≥80 years.

### Assessment and outcome definitions

The primary endpoint was major bleeding, and the secondary endpoint was the incidence of symptomatic VTE. Patients who met the inclusion criteria and received at least one dose of FPX were analyzed for these primary and secondary endpoints. Bleeding was classified as major if the event met at least one of the following definitions: fatal bleeding, retroperitoneal or intracranial bleeding, bleeding of critical organs (intraocular, adrenal, endocardial, or spinal bleeding), surgical site bleeding that required surgical intervention, or clinically overt bleeding with a decrease in hemoglobin (Hb) by at least 2 g/dl, or the need for transfusion of ≥800 ml red blood cells or whole blood. Minor bleeding was defined as bleeding that did not meet any of the major bleeding criteria.

If clinically suspicious symptoms of VTE were noted, such as dyspnea, chest pain, or decreased percutaneous arterial oxygen saturation (SpO_2_), enhanced multi-detector helical computed tomography (MDCT) with contrast media, pulmonary scintigraphy, or pulmonary arteriography was performed to look for PE. If there was lower extremity swelling, ultrasonography, MDCT, or ascending phlebography was done for the diagnosis of deep vein thrombosis. Primary and secondary endpoints were assessed during the period between when FPX was started and 1 day after its completion. Clinical symptoms as well as SpO_2_, plasma D-dimer, platelet count, and liver function were prospectively recorded preoperatively and on postoperative days (PODs) 1, 3, and 7.

### Statistical analysis

This trial was designed to demonstrate the safety of FPX in Japanese patients with colorectal cancer. Because we had no background data for patient recruitment, we referred to the APOLLO trial for the sample size calculation in terms of safety assessment [6]. Therefore, the recruitment target was set at 600 patients. All continuous data are expressed as the median (range). Frequency distributions between categorical data were compared using χ^2^ tests. The association between a major or minor bleeding event and the bleeding risk factors was assessed using multivariate logistic regression models. Results are expressed with odds ratios (ORs) and 95 % confidence intervals (CI). All statistical tests were two-sided, and all analyses were performed with SPSS 11.0J (IBM SPSS, Chicago, IL).

## Results

### Clinical characteristics of the study population

Between February 2009 and December 2010, 665 patients from 23 institutions were registered for this study, 619 (93.1 %) of whom met the inclusion criteria. These 619 patients received at least one dose of FPX and were included in the safety and efficacy analyses. The reasons for exclusion from the study included increased D-dimer (*n* = 23), no D-dimer values (*n* = 17), no histological evidence of malignancy (*n* = 5), and bleeding before FPX administration (*n* = 1). Table 1 shows the baseline clinical characteristics of the 619 patients and Table 2 summarizes the surgical procedures and related operational information. Two-hundred patients underwent open surgery and 419 patients underwent laparoscopic surgery, which was converted to open surgery in 27 (6.4 %).

**Table 1:** Background clinical characteristics of the patients (*n* = 619)

Age (years), mean (SD)	66.6 (9.5)
Sex (M/F)	371/248
Weight (kg), mean (SD)	57.8 (11.0)
BMI (kg/m^2^), mean (SD)	22.4 (3.3)
Diagnosis, *n* (%)	
Cancer	615 (99.4)
Carcinoid	4 (0.6)
Site of disease, *n* (%)	
Right-side colon	184 (29.7)
Left-side colon	192 (31.0)
Rectum	243 (39.3)

*BMI* body mass index (kg/m^2^)

**Table 2:** Operational procedure and surgical characteristics

	Open surgery (*n* = 200)	Laparoscopic surgery (*n* = 419)
Partial resection	5	21 (3)^a^
Ileocecal resection	11	40 (3)^a^
Right colectomy	38	70 (3)^a^
Left colectomy	10	28 (2)^a^
Sigmoidectomy	32	110 (6)^a^
Anterior resection	38	65 (3)^a^
Low anterior resection	48	82 (7)^a^
Abdominoperineal resection	12	2 (0)^a^
Total pelvic exenteration	1	1 (0)^a^
Subtotal colectomy	1	0
Colostomy	2	0
Trans-anal resection	1	0
Other	1	0
Operation time in minutes, median (range)	169.5 (30–651)	225 (72–586)
Blood loss in ml, median (range)	107.5 (0–7440)	25 (0–3635)
Use of epidural catheter, *n* (%)	170 (85)	107 (25.6)

^a^Values in parentheses indicate the number of laparoscopic procedures converted to open surgery

The mechanical prophylaxes used with FPX were as follows: EB for a median duration of 1 day (range 1–3 days) in 10 patients (1.6 %), 2 of whom received only EB; ES for a median duration of 1 day (range 0–7 days) in 518 patients (83.7 %); and IPC for a median duration of 0 days (mean 0.46 days, range 0–4 days) in 572 patients (92.4 %). In many institutions, IPC was discontinued after the patient began to ambulate on postoperative day (POD) 1 and ES were removed after the first injection of FPX. One patient did not receive any type of mechanical prophylaxis.

For pharmacological VTE prophylaxis, FPX was given at a dosage of 1.5 mg to 83 patients and at a dosage of 2.5 mg to 536 patients. The total median duration of FPX treatment at both 1.5 and 2.5 mg was 4 days (range 1–10 days).

### Safety outcomes

The incidence of major bleeding during the treatment period was 0.81 % (5/619) with a 95 % CI of 0.3–1.9 %. There was no death related to bleeding or from other causes during the treatment period. Table 3 summarizes the five cases of major bleeding. One patient with ascending colon cancer who underwent open surgery suffered a fall, sustaining a subdural hematoma after hitting his head against the floor. The other patient underwent low anterior resection as open surgery for rectal cancer. He exhibited an Hb decrease of greater than 2 g/dl and received an 800 ml transfusion for anemia 2 days after FPX administration. An upper gastrointestinal fiberscope revealed a bleeding ulcer in the gastric tube used for reconstruction after esophagectomy. Three patients from the laparoscopic surgery group suffered major bleeding at the anastomosis, after resection of ascending colon cancer in two patients and of descending colon cancer in one patient. All three patients had an Hb decrease >2 g/dl and one had concomitant anastomotic leakage necessitating re-operation.

**Table 3:** Occurrences of major bleeding

	Open surgery (*n* = 200)	Laparoscopic surgery (*n* = 419)	Total (*n* = 619)
	*n* (%)	*n* (%)	*n* (%)
Major bleeding	2 (1)	3 (0.72)	5 (0.81)
Fatal bleeding	0	0	0
Bleeding in a critical organ	1 (0.5)	0	1 (0.16)
Bleeding at the surgical site leading to re-operation	0	1 (0.24)	1 (0.16)
Bleeding at the surgical site with Hb decrease >2 g/dl	0	2 (0.48)	2 (0.32)
Bleeding at a non-surgical site with a Hb decrease >2 g/dl	1 (0.5)	0	1 (0.16)

*Hb* hemoglobin

The incidence of minor bleeding during the treatment period was 9.5 % (59/619). Most minor bleedings occurred at the surgical site, including the wound, the drain insertion site, and the anastomosis site (Table 4). Subcutaneous hemorrhage or hematoma was the most frequent event; followed by melena, caused mainly by bleeding of the anastomosis site. One patient had bleeding of the epidural catheter insertion site, but no subsequent symptoms of epidural hematoma were noted.

**Table 4:** Occurrences of minor bleeding

	Open surgery (*n* = 200)	Laparoscopic surgery (*n* = 419)	Total (*n* = 619)
	*n* (%)	*n* (%)	*n* (%)
Minor bleeding	12 (6.0)	47 (11.2)	59 (9.5)
Subcutaneous hemorrhage/hematoma	6 (3.0)	18 (4.3)	24 (3.9)
Melena anastomotic hemorrhage	2 (1.0)	21 (5.0)	23 (3.7)
Bloody drain discharge hemorrhage at drain site	4 (2.0)	6 (1.4)	10 (1.5)
Bleeding of gastric ulcer	0	1 (0.24)	1 (0.16)
Bleeding of epidural catheter insertion site	0	1 (0.24)	1 (0.16)

### Risk factors for bleeding events

To assess the risk factors for bleeding, univariable analysis was performed for major and minor bleeding events, and patient-related factors (age, sex, body weight, and BMI), surgery-related factors (mode of surgery, duration, and blood loss), FPX dose, and patient laboratory data (pre- and postoperative platelet count, D-dimer level, and liver function). Table 5 shows that sex (male), blood loss during surgery (<50 ml), preoperative platelet count (<15 × 10^4^/µl), and platelet count on POD 1 (<15 × 10^4^/µl) were associated with a significantly greater incidence of bleeding events. The threshold of the platelet count was defined as 15 × 10^4^/µl, being the lower limit of most institutional normal ranges of 13–14 × 10^4^/µl. We then performed multivariate analysis using factors with *p* values of <0.2, excluding the platelet count on POD 1. This revealed that a preoperative platelet count of <15 × 10^4^/µl, male sex, and intraoperative blood loss of less than 50 ml were independent risk factors.

**Table 5:** Univariable and multivariable analysis of factors associated with bleeding events

Factor	*n*	Incidence of bleeding events (%)	*p* value	Odds ratio	*p*	95 % CI
Age						
<80	572	10.3	1.000			
≥80	47	10.6				
Sex						
Male	371	12.7	0.022	2.078	0.016	1.143–3.778
Female	248	6.9		Reference		
Body weight (kg)						
≤40	19	15.8	0.434			
>40	600	10.2				
BMI (kg/m^2^)						
≤18	41	17.0	0.177	2.170	0.092	0.881–5.349
>18	578	9.9		Reference		
Surgery						
Open	200	7.0	0.067	Reference		
Laparoscopic	419	11.9		1.674	0.126	0.865–3.238
Operation time (min)						
<180	203	8.4	0.325			
≥180	416	11.3				
Blood loss (ml)						
<50	328	13.1	0.017	2.019	0.020	1.120–3.640
≥50	291	7.2		Reference		
FPX dose (mg)						
1.5	83	12.0	0.563			
2.5	536	10.1				
Pre-op D-dimer (µg/ml)						
<0.5	253	12.3	0.227			
≥0.5	366	9.0				
Pre-op platelet count (×10^4^/µl)						
<15	41	29.3	<0.0001	4.521	0000	2.081–9.822
≥15	578	9.0		Reference		
Platelet count on POD 1 (× 10^4^/µl)						
<15	109	16.5	0.023			
≥15	507	8.9				
Pre-op AST (U/L)						
≤40	582	10.5	0.781			
>40	36	11.1				
Pre-op ALT (U/L)						
≤40	583	9.9	0.162	Reference		
>40	35	17.1		1.628	0.336	0.603–4.398

*CI* confidence interval, *BMI* body mass index, *FPX* fondaparinux, *POD* postoperative day, *AST* aspartate amino transferase, *ALT* alanine transaminase

### Efficacy outcomes

There was no incidence of symptomatic VTE or fatal VTE in this study.

## Discussion

In this series of patients undergoing surgery for colorectal cancer, no fatal bleeding occurred, although the incidences of major and minor bleeding were 0.81 and 9.5 %, respectively. In the APOLLO trial comparing FPX + IPC with IPC alone, incidences of major and minor bleeding were 1.6 % (10/635) and 0.8 % (5/635), respectively [6]. In another study comparing FPX with dalteparin, there were two cases (0.1 %) of fatal bleeding and a 2.0 % incidence of bleeding necessitating reoperation or intervention, with an incidence of major bleeding of 3.4 % [7].

On evaluating other agents, a previous study on general surgery found incidences of major hemorrhage and wound hematoma of 3.2 and 6.1 %, respectively, in patients treated with unfractionated heparin prophylaxis [8]. In a report comparing enoxaparin and unfractionated heparin for the prevention of VTE in cancer surgery, incidences of major bleeding were 4.1 and 2.9 %, respectively, and those of minor bleeding were 14.6 and 14.3 % [9]. Taken together, in the current group of patients treated with FPX, the safety profile was comparable with those of these studies.

In evaluating the efficacy endpoint, we found no incidence of symptomatic VTE in these 619 patients. This incidence is comparable with those in the FPX prophylaxis arms of two previous studies, reporting 0.2 % (1/650) and 0.4 % (6/1465), respectively [6, 7].

We identified three randomized studies on the prevention of VTE in patients with colorectal surgery [10–12]. A randomized phase III trial reported incidences of 1.5 % (10/643) and 2.7 % (18/653) for major bleeding and 0.6 % (3/468) and 0.4 % (2/468) for symptomatic VTE, respectively, in patients receiving low-dose unfractionated heparin and enoxaparin [10]. Another phase III study compared nadroparin and enoxaparin in colorectal cancer surgery, and reported incidences of 7.3 % (47/643) and 11.5 % (72/628) for major bleeding and 0.2 % (1/643) and 1.4 % (9/628) for symptomatic VTE, respectively [11]. The high incidence of major bleeding in that study was attributed to the definition of blood loss during the operation, which was not included in the study. In Singapore, Ho et al. [12] investigated the efficacy of enoxaparin in colorectal surgery and found that the patients given enoxaparin prophylaxis vs. those not given prophylaxis had VTE incidences of 0 and 5 %, respectively. Bleeding events were more common in the enoxaparin prophylaxis group (6.7 %) than in the no-prophylaxis group (1.8 %), with three cases (2.2 %) of major bleeding events in the enoxaparin prophylaxis group. Considering these data on colorectal surgery, our present data demonstrate that VTE prophylaxis with FPX in patients with colorectal cancer is safe and effective.

Several randomized phase III trials of VTE prophylaxis have used pharmacological agents; however, the bleeding risk during pharmacological prophylaxis has rarely been analyzed [13]. This may be due to the fact that most studies include a wide variety of patient conditions. Because only patients with colorectal cancer were included in the present study, we sought to find risk factors for bleeding mainly in terms of patient-related and operational factors. We found that a preoperative platelet count <15 × 10^4^/µl, male sex, and bleeding <50 ml during the operation were independent risk factors for postoperative bleeding. Male sex was previously identified as a risk factor for bleeding in abdominal surgery as men have a small pelvic cavity rich in visceral fat, which makes hemostasis difficult [13, 14]. Moreover, in the Japanese population, being female is a risk factor for VTE in abdominal surgery [2], which may explain why women bleed less. It is not clear why less bleeding during the operation is a risk factor for postoperative bleeding. It is possible that a very small amount of bleeding will not induce sufficient natural coagulability to prevent postoperative hemorrhage. This rationale would also explain why laparoscopic surgery is associated with a lower rate of VTE [15, 16].

Of the five major bleeding events in this series, three were anastomotic bleeding, one of which was accompanied by anastomotic leakage and required re-operation. Those anastomoses were performed in a so-called “functional end-to-end” fashion using a mechanical stapler, in the right colon [17]. In this situation, precautions should be taken, especially on the first stapling of intraluminal mucosal edges. Any bleeding from the mucosal edges should be stopped by suturing or electro-coagulation. Bleeding events from this site tend to be major because they manifest more slowly than from left-side colon anastomoses.

In Japan, FPX and enoxaparin are used as VTE prophylaxis after abdominal high-risk surgery. It is very important to know which agent is safer, but there is no evidence to distinguish these two agents in terms of their safety profile. In comparison with enoxaparin, FPX has a longer half-life and no antidote [18], so it is given once a day, and if bleeding occurs, all we can do is to stop its administration. The fact that all bleeding events in this series were controlled by stopping FPX demonstrates that its prompt discontinuation is necessary in the case of bleeding.

The weaknesses of this study are that there was no control group, the duration of VTE prophylaxis was only 4–8 days, and the observation period was only up until POD 5. Without a control arm, the incidence of bleeding and symptomatic VTE will be descriptive; however, more than 600 patients is sufficient to evaluate the safety of FPX. Because the ENOXACAN II study showed that 4 weeks of enoxaparin prophylaxis significantly reduced the incidence of venographically detected thrombosis, longer prophylaxis seems warranted [19]. However, only 4–8 days of FPX is approved by the government in Japan, and the observational period of this study was thus necessarily short. We plan to evaluate the usefulness of longer prophylaxis in the next trial.

In conclusion, thromboprophylaxis in cancer patients is complicated by the fact that they are at increased risk of both VTE and bleeding [20]. Thus, it is important to identify the best way to prevent thrombosis while minimizing bleeding complications. Ample information about the bleeding risks of specific surgical procedures may help surgeons use pharmacological prophylaxis more effectively. The findings of the present study suggest that FPX given once daily at a dose of 2.5 mg, initiated 24 ± 2 h after an operation, is safe and effective for Japanese patients undergoing colorectal cancer surgery.

## Appendix

The following institutions and investigators participated in this study, in no particular order.

Kimimasa Ikeda and Masakazu Miyake, Toyonaka Municipal Hospital; Hideyuki Mishima and Masakazu Ikenaga, Osaka National Hospital; Yoshihito Ide, Suita Municipal Hospital; Kazuya Sakata, Higashiosaka City General Hospital; Shingo Noura and Tatsushi Shingai, Osaka Medical Center for Cancer and Cardiovascular Diseases; Hirofumi Yamamoto, Ichiro Takemasa, Junichi Nishimura, and Mamoru Uemura, Osaka University Hospital; Hiroki Akamatsu, Osaka Police Hospital; Chu Matsuda, Osaka General Medical Center; Keigo Yasumasa, Osaka Kouseinenkin Hospital; Atsushi Ohkawa, Higashi Takarazuka Satoh Hospital; Shunji Morita, Yao Municipal Hospital; Hitoshi Mizuno, Rinku General Medical Center; Yasunori Watanabe, Osaka Sen-in Hospital; Seiji Kawasaki, Kobe Ekisaikai Hospital; Osamu Takayama, Itami City Hospital; Masato Sakon, Takayuki Ichihara, and Masakazu Murakami, Nishinomiya Municipal Central Hospital; Yasuhiro Miyake, Minoh City Hospital; Takamichi Komori and Yoshio Uemura, Kinki Central Hospital of Mutual Aid Association of Public Teachers; Mutsumi Fukunaga and Hiroyoshi Takemoto, Sakai City Hospital.
